# Milk production and composition in warm-climate regions: a systematic review and meta-analysis

**DOI:** 10.1007/s11250-024-04214-5

**Published:** 2024-11-14

**Authors:** Mohamed Rashid, Hadeer M. Aboshady, Rania Agamy, Harry Archimede

**Affiliations:** 1 Regional Center for Food and Feed, Agricultural Research Center, Giza, 12619 Egypt; 2 Animal Production Department, Faculty of Agriculture, Cairo University, Giza, 12613 Egypt; 3 Agroecology, Genetic and Tropical Livestock Farming System, INRAE, Petit-Bourg, Guadeloupe 97170 France

**Keywords:** Meta-analysis, Milk production, Milk constitute, Milk fat, Milk protein, Lactating ruminant, Warm-climate regions, Tropics

## Abstract

Milk production is a key component of the agriculture sector in the tropics and subtropics, contributing 47.32% to global milk production. This study aimed to quantify milk production and composition (fat and protein) in warm-climate regions located between 30 degrees north and south of the equator. A meta-analysis was conducted using the standardized mean (SM) for milk production, fat percentage, and protein percentage, all adjusted for dry matter intake (DMI), focusing on lactating ruminants. A total of 42, 11, 15, and 16 research papers were selected for cows, buffalo, sheep, and goats, respectively, encompassing 2421 animal records from experiments published between 1992 and 2024. The SM for milk production was 10.38, 9.77, 0.79, and 1.13 kg/day/animal for cows, buffalo, sheep, and goats, respectively. Due to the significant variance between different cow breeds, the study divided the cows into three main groups based on breed type crossbreds, foreign, and local breeds. The SM for milk production per animal was 11.49 kg/day for crossbreds, 22.95 kg/day for foreign breeds, and 7.13 kg/day for local breeds. The effect of DMI on the SM of milk production for cows, sheep, and goats was highly significant. For milk fat, the SM was 3.95, 6.64, 4.70, and 3.56% for cows, buffalo, sheep, and goats, respectively. Regarding milk protein, the SM was 3.36, 3.91, 4.34, and 3.45% for cows, buffalo, sheep, and goats, respectively. The results of this meta-analysis highlight that warm-climate regions are significant contributors to global dairy production. Furthermore, improving ruminant milk production and quality in hot climates need further efforts.

## Introduction

The world’s climates are incredibly diverse; based on factors like temperature, humidity, wind, radiation, and precipitation patterns, they can be broadly categorized into several major types. Tropical and subtropical climates, both characterized by consistently high temperatures throughout the year, play a significant role in shaping ecosystems and influencing the distribution of both animal and plant species (Johnson 1976). The temperature-humidity index (THI) is a common indicator used to assess the degree of heat stress, taking into account thermal conditions and relative humidity (Lambertz et al. 2014).

Several factors influence animal milk yield and composition, including animal breed, genetic factors, season, feeding strategy, stage of lactation, parity, milking frequency, and the animal’s health status (Toghdory et al. 2022). In tropical and subtropical regions, where the climate is consistently hot and humid, ruminants are raised for both meat and milk production while being exposed to higher ambient temperatures, humidity, and solar radiation (Habimana et al. 2023). These environmental factors affect milk yield and composition, including fat, protein, lactose, solids-not-fat percentages, and somatic cell count (Dunn et al. 2014; Lambertz et al. 2014). Moreover, an increase in the THI exacerbates the presence of pathogens in the environment, leading to a higher occurrence of mastitis and an increased milk microbial load (Toghdory et al. 2022).

The thermo-neutral zone of lactating animals ranges from 16℃ to 25℃; within this range, the animals maintained their body temperature between 38.4–39.1℃ (Das et al. 2016). In warm-climates, due to the high temperature affecting the animal’s body surface, there are several physiological responses, including an increase in respiration rate, heart rate, and rectal temperature. These physiological changes directly impact the performance of lactating animals, especially those with high genetic merit for milk production, leading to increased water intake and decreased feed intake, milk production, and reproductive performance (Cowley et al. 2015).

In the tropics, animal feeding strategies primarily involve grazing on locally regrown grass. Smallholder farmers predominantly use pasture as a feed source for lactating animals due to its low production cost (Rosendo-Ponce et al. 2021). Supplementing animals’ diets with concentrated feed mixtures is both profitable and efficient for increasing milk production (Clark et al. 2018). For example, in a temperate climates and under grazing conditions, the milk production of lactating Holstein cattle doubled with the addition of a commercial feed supplement (Clark et al. 2018).

Dairy breeds in tropical and subtropical regions generally mature slowly and produce low amounts of milk. This is partly due to malnutrition, poor management, and challenging environmental conditions (Das et al. 2003). While local breeds in these regions are well adapted to environmental stresses, their milk production remains very low (Usman et al. 2013). Consequently, these low-producing local breeds are typically raised under grazing systems (Leeuw et al. 1998; Seré and Steinfeld 1996). Throughout the Caribbean, Latin America and sub-Saharan Africa, sheep and goats are commonly rare under small scale systems, family based, where animals usually kept as a component in mixed farming practices (Ben Salem et al. 2004).

For cattle, Holstein Friesian is the highest milk-producing dairy breed, originally developed in temperate zones of developed countries. Many developing countries in tropical and subtropical regions import Holstein Friesians, either to maintain them as a pure breed or to crossbreed them with local low-producing cattle breeds (Usman et al. 2013). In tropical and subtropical countries, these specialized temperate dairy breeds are typically kept under intensive, semi-controlled environments due to their high genetic potential for milk production and sensitivity to harsh tropical conditions (Usman et al. 2013). When Holstein Friesians are crossbred with local low-producing cattle in these regions, the resulting crossbreeds combine moderate to high milk production with better adaptability to harsh environments. These crossbred animals are usually raised under intensive and semi-intensive systems.

However, Rosendo-Ponce et al. (2021) found that supplementing crossbreed cows’ diet with commercial feed (3.5 and 7.0 kg/cow/d) under grazing conditions in tropical climates did not significantly increase milk production. This finding suggests that response of grazing animals to supplementation with concentrated feed mixtures is influenced by breed genetic. Crossbreeding local animals with temperate dairy breeds is commonly recommended to achieve acceptable productivity and resilience in hot local climates. local breeds typically maintain good health and productive performance, being well-adapted to the local climate, feed resources, and management systems (Buaban et al. 2015).

Tropical dairy farming is a remarkably efficient system that can convert abundant tropical forages into highly nutritious milk, considered one of the most wholesome human foods (Moti et al. 2020). However, in many tropical countries, locally produced milk is insufficient to achieve self-sufficiency. Constraints on dairy farms include adverse weather conditions (high THI), which affect animal production, as well as issues related to diseases and internal parasites. Additionally, young animals are often raised in smallholder systems with low inputs and minimum management. Another significant constraint is that most animal breeds in tropical countries are multi-purpose breeds with low genetic potential for milk production.

Tropical and subtropical regions exhibit varying temperatures and humidity levels. For example, in Sri Lanka, the average temperature ranges between 28.0℃ and 32.0℃, with THI values varying from 68.57 to 83.17 across different parts of the country (Weerasinghe et al. 2024). In Cuba, the THI fluctuates throughout the day, starting at 69.59 in the morning, rising to 79.61 at midday, and reaching 91.69 in the afternoon (Valdivia-Cruz et al. 2021). Similarly, in the tropical region of India (Palakkad), the maximum daytime temperature ranges between 32.27℃ and 41.05℃, with average relative humidity varying from 44.1 to 92.3% between January and March, and THI values ranging from 71.88 to 83.21 (Pramod et al. 2021). In Tanzania, THI values were recorded within a wide range, from 61 to 86 (Ekine-Dzivenu et al. 2020).

Livestock species generally experience no distress at a THI of 65–72. Mild stress occurs when the THI is between 72 and 78, while severe stress is felt when the THI exceeds 80 (Habib et al. 2020). As homeotherms, dairy livestock experience stress when exposed to sudden and extreme fluctuations in environmental temperatures. For bovines, the thermal comfort zone is maintained within a body temperature range of 36.7 °C to 39.1 °C (Seeram 2020). Hot and humid conditions in tropical and subtropical climates can adversely impact the health and productivity of dairy animals (Singh et al. 2020). When the environmental temperature rises above 38℃, animals experience thermoregulation failure, leading to elevated rectal temperatures and respiratory rates, as well as reduced feed intake and milk production (Ekine-Dzivenu et al. 2020).

To the best of our knowledge, there are a limited number of reviews on lactating ruminant production and milk composition in the tropics and subtropics. This review focuses on the characteristics of ruminant milk production and composition in warm-climate countries.

## Materials and methods

### Literature search

A literature search was conducted in Scopus, PubMed, Web of Science, and Google Scholar, along with an examination of citations in scientific papers. The selected studies had to meet the following criteria: (1) the location of the experimental trials needed to be in warm-climate regions, such as Africa, Australia, South Asia, the Caribbean, Central America, and South America. The tropics are commonly defined as the region between the Tropic of Cancer (approximately 23.5 degrees North latitude) and the Tropic of Capricorn (approximately 23.5 degrees South latitude), highlighted in crimson. For our purposes, we extended the definition of the tropics to include the area from 30 degrees North to 30 degrees South latitude; (2) lactating animals (cows, buffaloes, sheep, or goats) had to be used as experimental units; (3) the studies had to report information on dry matter intake (DMI), milk production, and milk fat and protein; (4) the studies had to report the standard error or standard deviation of the mean for the measured variables. After applying the selection criteria to approximately one hundred and twenty studies, 42, 11, 15, and 16 publications were selected for cows, buffalo, sheep, and goats, respectively, as they met the criteria for inclusion in the meta-analysis (Table 1).

**Table 1:** List of the studies included in the systematic review and meta-analysis

References	Regions	Species
(Abd El Tawab et al. 2017)	Egypt	Cattle
(Ajmal Khan et al. 2004)	Pakistan	Cattle
(Aung et al. 2016)	Myanmar	Cattle
(Barletta et al. 2016)	Brazil	Cattle
(Barwani et al. 2023)	Congo	Cattle
(Bhatta et al. 2000)	India	Cattle
(Boyd et al. 2013)	USA (Tifton)	Cattle
(Carmo et al. 2014)	Brazil	Cattle
(Castro-Montoya et al. 2018)	El Salvador	Cattle
(Dey et al. 2009)	India	Cattle
(Dey et al. 2017)	India	Cattle
(Dey and De 2014)	India	Cattle
(Dos Santos et al. 2015)	Brazil	Cattle
(Ferreira et al. 2017)	Brazil	Cattle
(Gado et al. 2009)	Egypt	Cattle
(Gebreyowhans and Zegeye 2019)	Ethiopia	Cattle
(Gunun et al. 2019)	Thailand	Cattle
(Imaizumi et al. 2016)	Brazil	Cattle
(Kumar et al. 2005)	India	Cattle
(Matloup et al. 2017)	Egypt	Cattle
(Mendieta-Araica et al. 2011)	Nicaragua	Cattle
(Mohanta et al. 2010)	India	Cattle
(Polyorach et al. 2015)	Thailand	Cattle
(Reyes Sánchez et al. 2006)	Nicaragua	Cattle
(Rodrigues et al. 2017)	Brazil	Cattle
(Rodrigues et al. 2018)	Brazil	Cattle
(Ruiz et al. 1992)	USA (Florida)	Cattle
(Sarwar et al. 2003)	Pakistan	Cattle
(Sidibe-Anago et al. 2006)	Burkina Faso	Cattle
(Vazirigohar et al. 2014)	Iran	Cattle
(Wanapat et al. 2018a, b)	Thailand	Cattle
(Zeng et al. 2018)	China	Cattle
(Zhu et al. 2016)	China	Cattle
(Signor et al. 2024)	Brazil	Cattle
(Abo-Donia et al. 2024)	Egypt	Cattle
(Galvão et al. 2024)	Brazil	Cattle
(Moura et al. 2023)	Brazil	Cattle
(Varela et al. 2023)	Brazil	Cattle
(Flórez-Delgado et al. 2023)	Colombia	Cattle
(Durman et al. 2022)	Brazil	Cattle
(Vieyra-Alberto et al. 2022)	Mexico	Cattle
(Andrade et al. 2023)	Brazil	Cattle
(Abo-Donia et al. 2022)	Egypt	Buffalo
(Ajmal Khan et al. 2006a)	Pakistan	Buffalo
(Ajmal Khan et al. 2006b)	Pakistan	Buffalo
(Hanafy et al. 2009)	Egypt	Buffalo
(Hussain et al. 2023)	Pakistan	Buffalo
(Morsy et al. 2016)	Egypt	Buffalo
(Nisa et al. 2004)	Pakistan	Buffalo
(Saleem et al. 2018)	Egypt	Buffalo
(Sarwar et al. 2004)	Pakistan	Buffalo
(Sarwar et al. 2005)	Pakistan	Buffalo
(Touqir et al. 2007)	Pakistan	Buffalo
(Aloueedat et al. 2019)	Jordan	Sheep
(Awawdeh et al. 2009)	Jordan	Sheep
(El-Gindy et al. 2021)	Egypt	Sheep
(Hadhoud et al. 2021)	Egypt	Sheep
(Khalil et al. 2023)	Egypt	Sheep
(Khattab et al. 2020)	Egypt	Sheep
(Mirheidari et al. 2019)	Iran	Sheep
(Morshedy et al. 2020)	Egypt	Sheep
(Obeidat 2023)	Jordan	Sheep
(Obeidat et al. 2010)	Jordan	Sheep
(Obeidat et al. 2011)	Jordan	Sheep
(Obeidat et al. 2014)	Jordan	Sheep
(Obeidat and Shdaifat 2013)	Jordan	Sheep
(Peniche-Gonzalez et al. 2018)	Mexico	Sheep
(Shdaifat et al. 2013)	Jordan	Sheep
(El-Zaiat et al. 2018)	Egypt	Goats
(El-Zaiat et al. 2022)	Egypt	Goats
(Ghoneem et al. 2015)	Egypt	Goats
(Ghoneem and El-Tanany 2023)	Egypt	Goats
(Gouda et al. 2019)	Egypt	Goats
(Hussein et al. 2022)	Egypt	Goats
(Khattab et al. 2011)	Egypt	Goats
(Kholif et al. 2018)	Egypt	Goats
(Kholif et al. 2017a, b)	Egypt	Goats
(Kholif et al. 2014)	Egypt	Goats
(Kholif et al. 2015)	Egypt	Goats
(Kholif et al. 2016)	Egypt	Goats
(Kholif et al. 2017)	Egypt	Goats
(Morsy et al. 2018)	Egypt	Goats
(Rojo et al. 2015)	Mexico	Goats
(Sahoo and Walli 2008)	India	Goats

### Parameters and data extraction

The parameters evaluated were DMI, milk production, and the percentages of milk fat and protein. The data set included the mean, standard deviation, and number of cows per treatment group. The precision of the estimate was based on the standard deviation for treatment and control groups as reported in the articles. If an article did not provide the standard deviation, it was calculated by multiplying the standard error of the mean by the square root of the number of animals.

### Statistical analyses

All statistical analyses and figures were performed using the statistical software package R 4.3.0 (R Core Team 2014). The milk production and composition of lactating dairy cows, buffalo, sheep, and goats were evaluated using a general linear (mixed-effects) model via the Metafor package in R. First, the relevant results from each study were quantified to compute the effect size (ES) so that the resulting values could be aggregated and compared. The ES was calculated based on the number of animals used in each study, with a 95% confidence interval.

A restricted maximum likelihood (REML) estimation model was used, the DMI included as a moderator to adjust for variation among studies for each variable (milk production, fat, and protein percentages). Finally, a meta-regression model was applied to predict the average mean of each variable. Statistical significance was considered at *P* ≤ 0.05. The meta-regression model was as following:

$$\:{y}_{ij}=\mu\:+{v}_{i}+\beta\:{x}_{ij}+{e}_{ij}$$

Where $\:{y}_{ij}$ is the dependent variable (production trait), µ is the overall mean (intercept), $\:{v}_{i}$ is a random effect of the i ^th^ study describing the study-specific deviation from the distribution mean, β is the regression coefficient of Y on X, $\:{x}_{ij}$ is the value of the continuous predictor variable (DMI), and $\:{e}_{i}$ is a random error term describing sampling variability. In addition, for cattle model a factor for breed type was included in the model with 3 levels (1: crossbred, 2: foreign breed and 3: local breed). In this context, local breeds is usually used as multipurpose animals and raised in extensive system, foreign breeds usually specialized and raised in intensive system, while crossbred is usually raised under semi-intensive systems).

## Results

### Contribution of warm-climate regions to global milk production

**Fig. 1:**
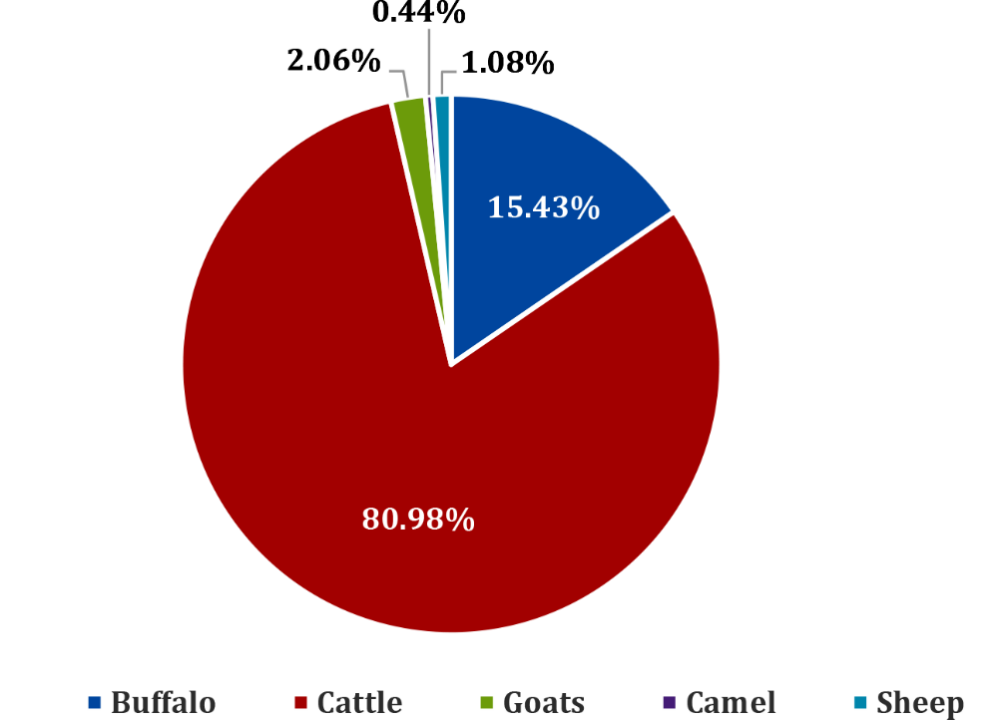
The percentage of participation of cattle, buffalo, camel, goat, and sheep in global milk production in 2022 developed from FAOSTAT (2024) dataset

According to FAOSTAT (2024), global milk production from cattle, buffalo, camels, goats, and sheep in 2022 was recorded at 930.30 million tons. Figure 1 shows the percentage contribution of each species in global milk production in 2022. The warm-climate regions (Africa, Australia, South Asia, the Caribbean, Central America, and South America) contributed 47.32% to the total world milk production. Among lactating ruminant species, dairy cattle produced 753.32 million tons of milk worldwide, representing approximately 80.98% of the total milk production. Buffalo milk is the second most produced type, accounting for 15.43% of the total milk production. Additionally, goats and sheep contributed 2.06% and 1.08% to global milk production, respectively. Camel milk recorded the lowest percentage, making up only about 0.44% of the world’s milk production.

**Fig. 2:**
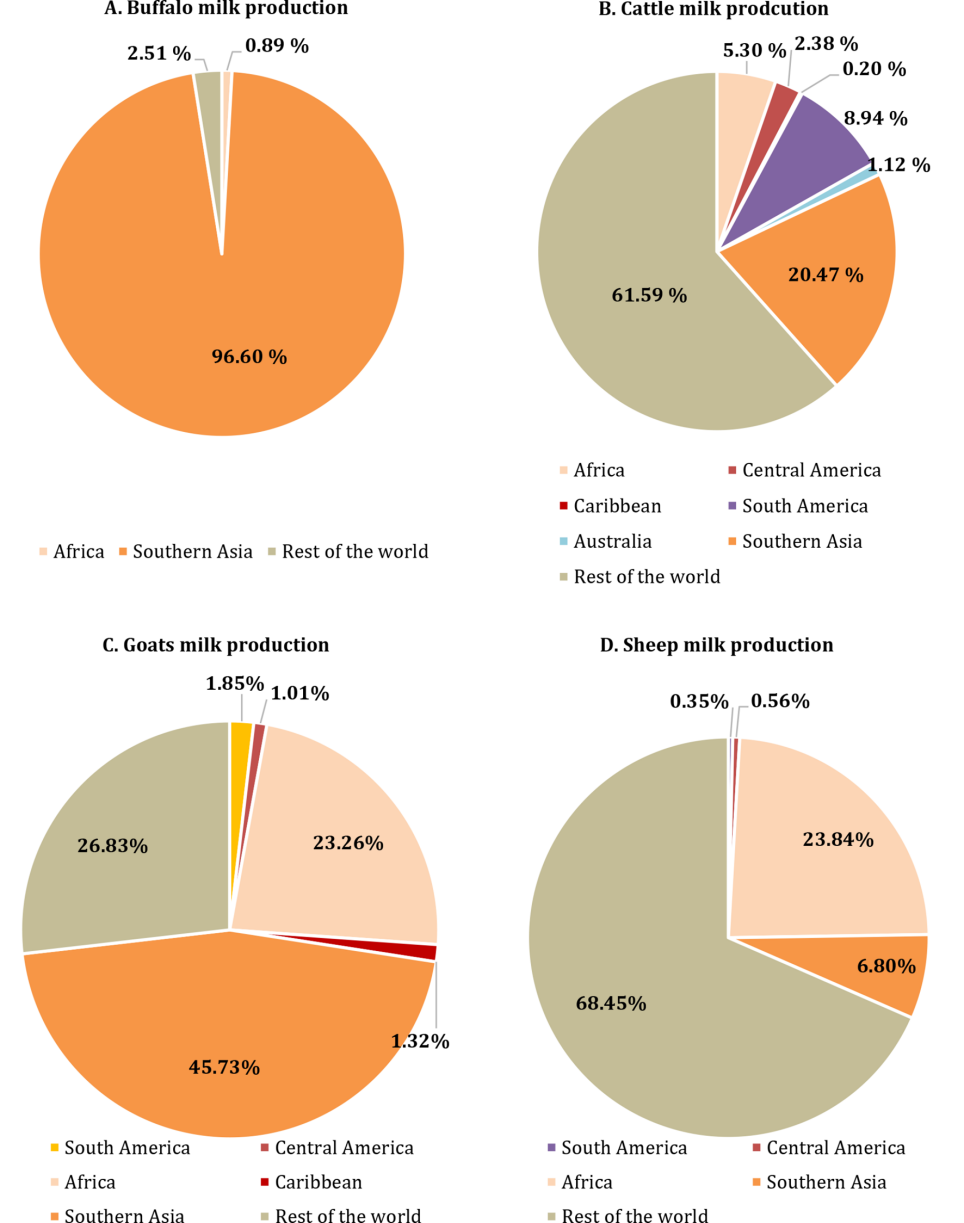
The percentage of participation in world milk production by various dominant lactating species (cattle, buffalo, goats, and sheep) in warm-climate countries (Africa, Australia, South Asia, the Caribbean, Central America, and South America) in 2022 developed from FAOSTAT (2024) dataset

Figure 2 illustrates the percentage contribution of various ruminant mammals (cattle, buffalo, goat, and sheep) to global milk production in warm-climate regions, including Africa, Australia, Southern Asia, the Caribbean, Central America, and South America, in 2022. Cattle in tropical and subtropical regions account for 38.41% of the world’s total cattle milk production, with Southern Asia, South America, and Africa contributing 20.47%, 8.94%, and 5.30%, respectively. Cattle play a significant role in the agricultural economy and nutritional security of countries in South Asia, South America, and Africa. South Asia, in particular, is rich in bovine genetic resources, boasting over 200 indigenous cattle and buffalo breeds, each with distinct phenotypic and adaptive traits (Panigrahi et al. 2022).

Approximately 97.50% of global buffalo milk production is produced by warm-climate countries, with Southern Asia alone contributes 96.60% of total world buffalo milk production (FAOSTAT 2024). About 98.0% of the world’s buffalo population is found in Asia, while 0.8% resides in Africa. Among tropical countries, India, Pakistan, China, Nepal, and Egypt have the highest populations of dairy water buffalo (Tsuji et al. 2022). Buffaloes are recognized as highly efficient converters of low-quality forage and agricultural by-products into high-quality milk. consequently, smallholder farmers often keep buffalo in mixed crop-livestock systems (Windsor et al. 2021).

Goat milk production is primarily concentrated in hot-climate countries, accounting for 73.17% of total goat milk production. This concentration is strongly influenced by sociocultural factors and limited resource availability. In such context, small ruminants are particularly promising due to their lower production costs, shorter generation intervals, suitability for smallholdings, and multipurpose roles (meat, milk, and fiber production) (Gama Pantoja et al. 2022). They are also highly effective in utilizing crop residues and, importantly, exhibit greater tolerance to harsh climatic conditions such as low rainfall and heat stress compared to cattle (Panigrahi et al. 2022).

The milk production of tropical sheep breeds, such as hair breeds, is typically sufficient only for the growth of lambs. Therefore, sheep farming in the tropics is primarily focused on meat production. Among ruminant species, sheep are the best suited for efficiently utilizing scarce vegetation in challenging and marginal areas through rangeland management. Tropical countries contribute approximately 31.55% to global sheep milk production, with Africa and Southern Asia being the top contributors, accounting for 23.84% and 6.80% of the total world sheep milk production, respectively.

### Cows’ milk production and composition under warm-climates

**Fig. 3:**
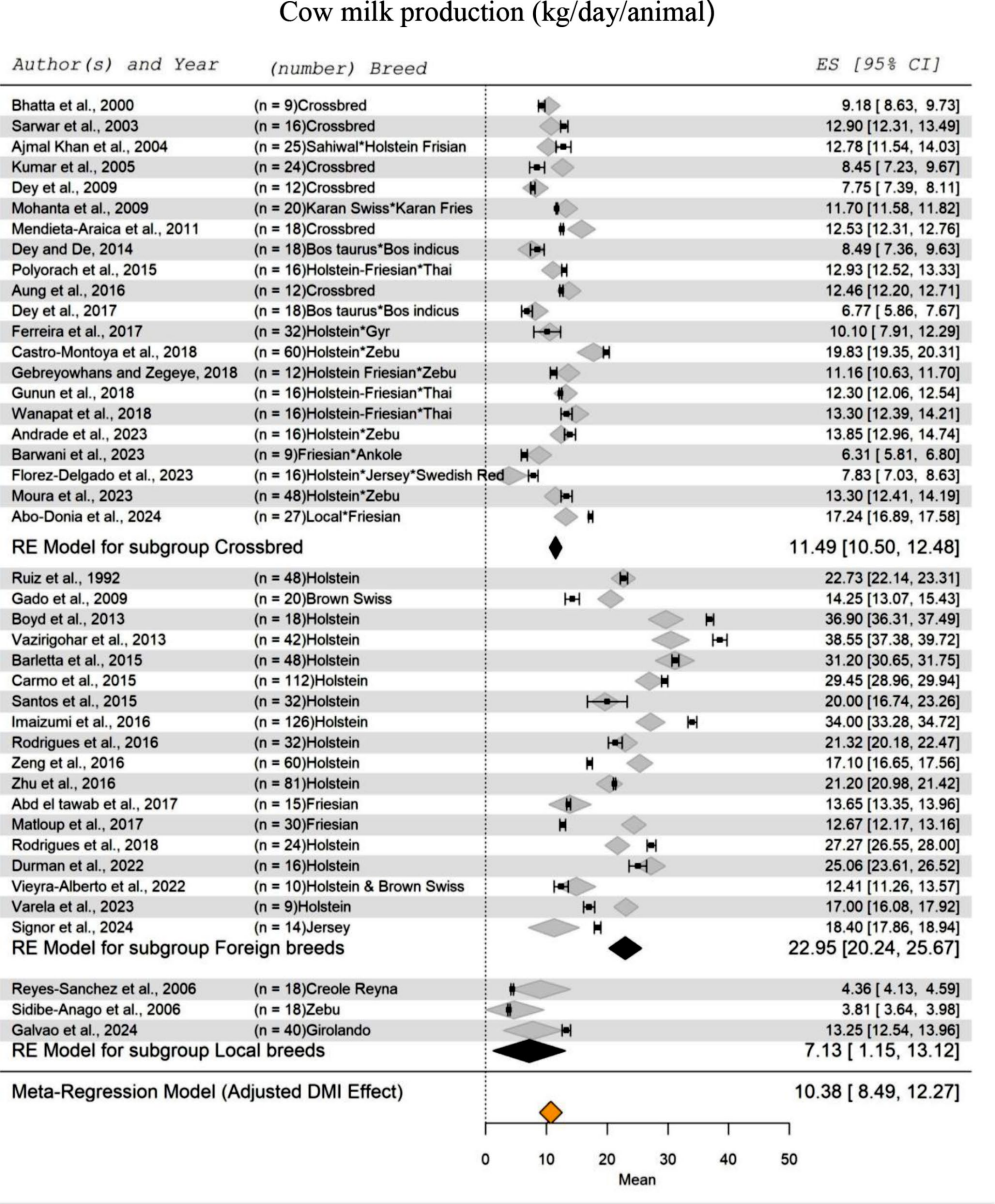
Forest Plot for Meta-Regression Analysis of milk yield (kg/day) in lactating cows with three subgroups (crossbreeds, foreign breeds, and local breeds). ‘n’ represents the number of animals used in each study, followed by the animal breed. The x-axis displays the z-statistic, which is the standardized mean (SM) for milk production. The length of the horizontal lines represents the 95% confidence interval for the SM of milk production. The gray diamond represents the adjusted milk production to dry matter intake (DMI). The effect size (ES) value is the estimated milk production mean corrected for the number of animals used in each study, with a 95% confidence interval between practices

A meta-analysis of multiple studies (Abd El Tawab et al., 2017; Abo-Donia et al. 2024; Ajmal Khan et al. 2004; Andrade et al. 2023; Aung et al. 2016; Barletta et al. 2016; Barwani et al. 2023; Bhatta et al. 2000; Boyd et al. 2013; Carmo et al. 2014; Castro-Montoya et al. 2018; Dey et al. 2009, 2017; Dey and De 2014; dos Santos et al. 2015; Durman et al. 2022; Ferreira et al. 2017; Flórez-Delgado et al. 2023; Gado et al. 2009; Galvão et al. 2024; Gebreyowhans and Zegeye 2019; Gunun et al. 2019; Imaizumi et al. 2016; Kumar et al. 2005; Matloup et al. 2017; Mendieta-Araica et al. 2011; Mohanta et al. 2010; Moura et al. 2023; Polyorach et al. 2015; Reyes Sánchez et al. 2006; Rodrigues et al. 2017, 2018; Ruiz et al. 1992; Sarwar et al. 2003; Sidibe-Anago et al. 2006; Signor et al. 2024; Varela et al. 2023; Vazirigohar et al. 2014; Vieyra-Alberto et al. 2022; Wanapat et al. 2018; Zeng et al. 2018; Zhu et al. 2016) on cow’s milk production in warm-climate regions is presented in forest plots (Fig. 3). The effect of DMI on the standardized mean (SM) of milk production was highly significant (*P* < 0.001). According to the research papers included in this meta-analysis, twenty-one studies involved crossbred cows, representing a total of 440 cow records. The SM of milk production for the crossbred cows ranged from 10.50 to 12.48 kg/day/cow, with an SM value of 11.49 kg/day/cow. Eighteen studies on foreign breeds raised in warm-climates were included, involving a total of 715 cow records. These foreign cow breeds, adapted to tropical and subtropical climates, recorded the highest milk production with an SM of 22.95 kg/day/cow. The standardized range for milk production ranged from 20.24 to 25.67 kg/day/cow, representing a 200% increase compared to the crossbreeds. The SM of milk production for the local breeds was 7.13 kg/day/cow, with a standardized range from 1.15 to 13.12 kg/day/cow. Overall, the meta-regression analysis showed that the general SM for cow milk production in warm-climate regions was 10.38 kg/d/cow, with a range from 8.49 to 12.27 kg/d/cow.

**Fig. 4:**
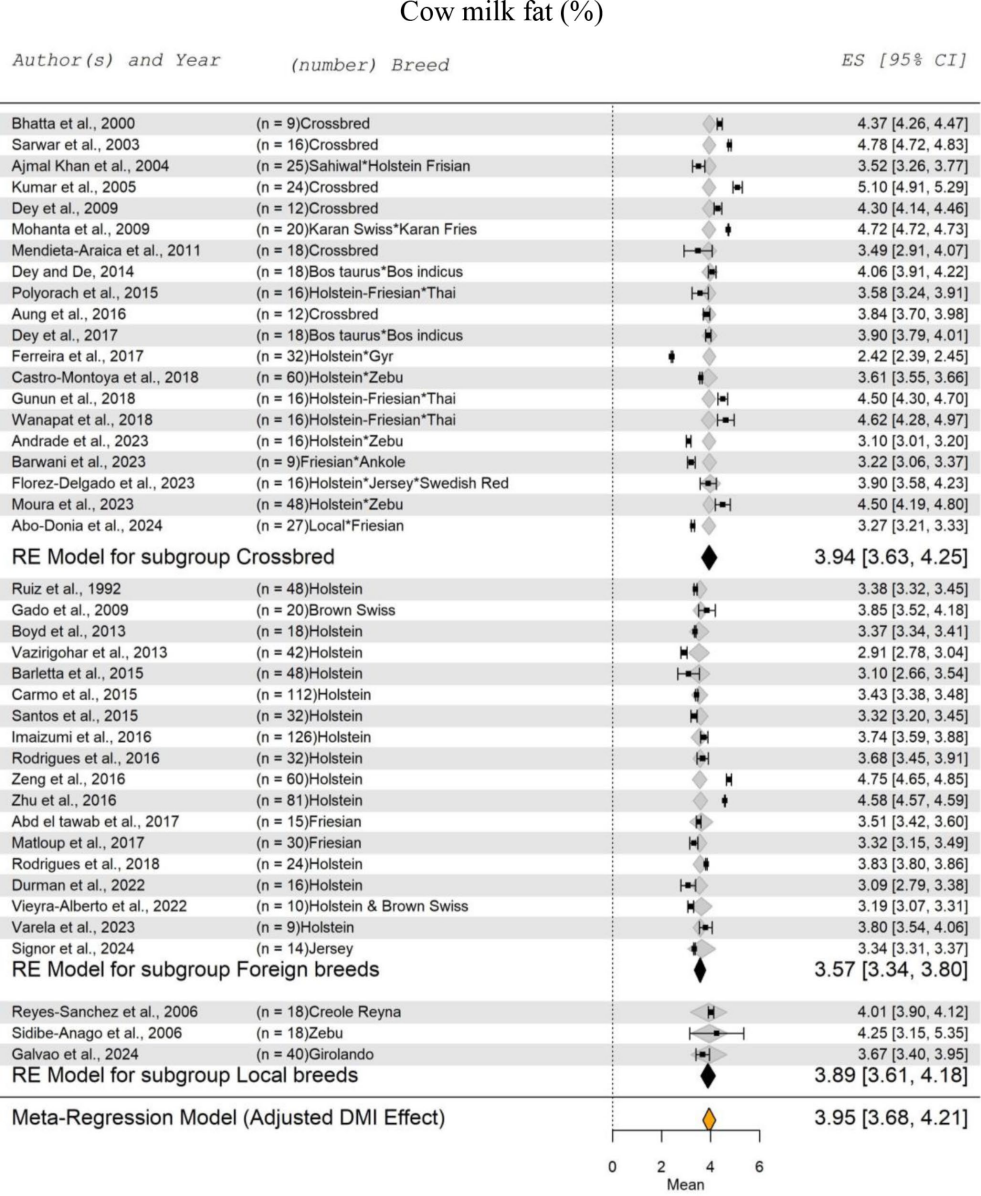
Forest Plot for Meta-Regression Analysis of milk fat% in lactating cows with three subgroups (crossbreeds, foreign breeds, and local breeds). ‘n’ represents the number of animals used in each study, followed by the animal breed. The x-axis displays the z-statistic, which is the standardized mean (SM) for fat%. The length of the horizontal lines represents the 95% confidence interval for the SM of fat%. The gray diamond represents the adjusted fat% to dry matter intake (DMI). The effect size (ES) value is the estimated fat% mean corrected for the number of animals used in each study, with a 95% confidence interval between practices

Figure 4 presents the forest plots of the meta-regression analysis for cow milk fat, adjusted for the DMI effect. The analysis included 41 studies, encompassing a total of 1241 cow records. The effect of DMI on the SM of milk fat was not significant (*P* = 0.7893). The SM of fat percentage for crossbred cow milk was recorded at 3.94%, with a range from 3.63 to 4.25%. For foreign breeds raised in warm-climates, the SM of milk fat was recorded at 3.57%, approximately 9.50% lower than that of crossbred cows. A similar trend was observed for native cow breeds, with an SM of milk fat at 3.89% and a range from 3.61 to 4.18%. The SM of fat percentage for local cow breeds was 8.50% higher than that of foreign breeds raised in warm-climates. Overall, regardless of breed, the SM for cow milk fat in tropical and subtropical regions was 3.95%, with a range from 3.68 to 4.21%.

**Fig. 5:**
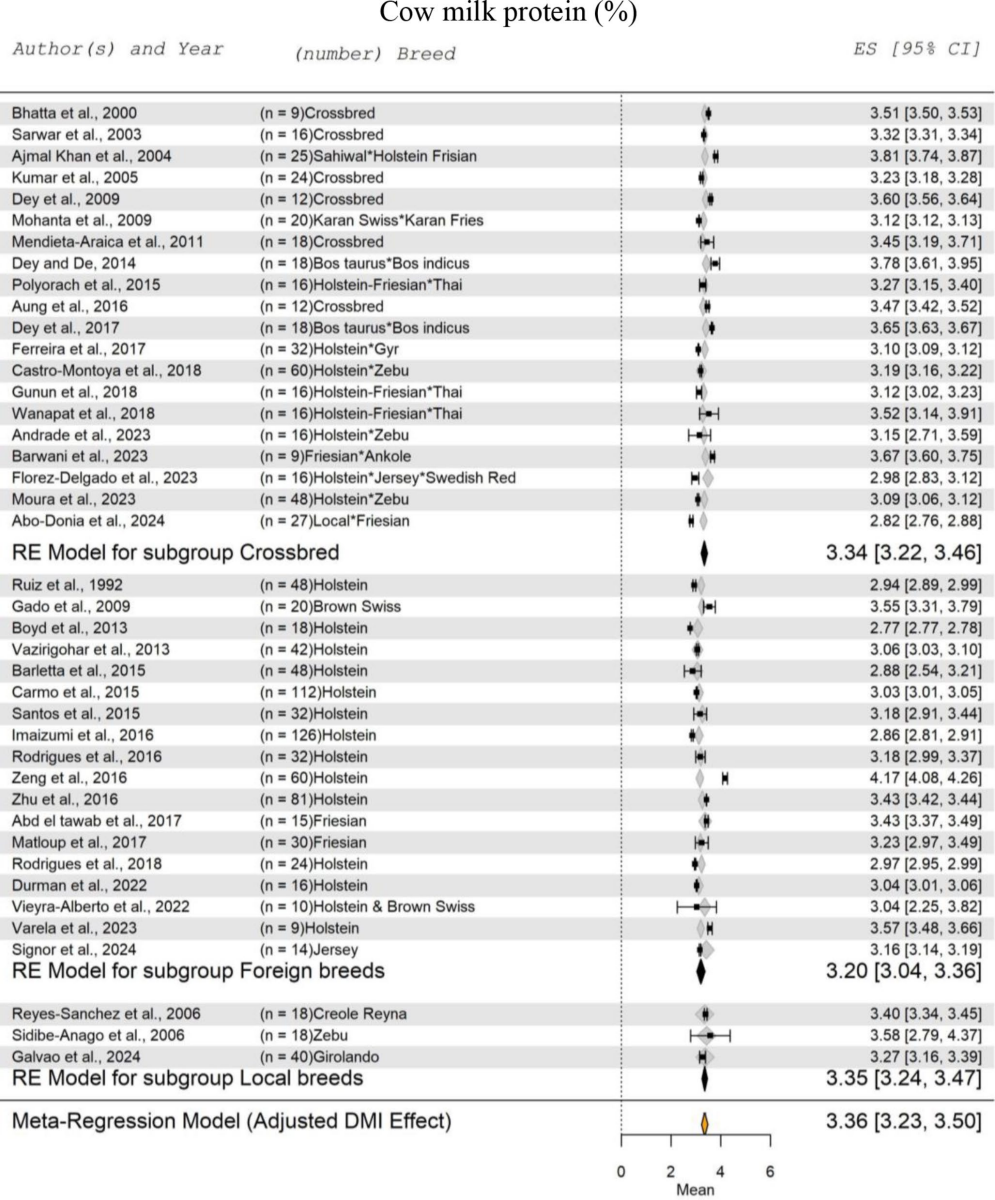
Forest Plot for Meta-Regression Analysis of milk protein% in lactating cows with three subgroups (crossbreeds, foreign breeds, and local breeds). ‘n’ represents the number of animals used in each study, followed by the animal breed. The x-axis displays the z-statistic, which is the standardized mean (SM) for protein%. The length of the horizontal lines represents the 95% confidence interval for the SM of protein%. The gray diamond represents the adjusted milk protein% to dry matter intake (DMI). The effect size (ES) value is the estimated protein% mean corrected for the number of animals used in each study, with a 95% confidence interval between practices

Figure 5 shows the meta-regression analysis for cow milk protein, adjusted for the effect of DMI. The analysis included 41 studies, encompassing a total of 1241 cow records. The effect of DMI on the SM of milk protein was insignificant (*P* = 0.1004). The highest SM for milk protein was recorded for local cow breeds, with a subgroup mean of 3.35%, ranging from 3.24 to 3.47%. In comparison, foreign breeds recorded a SM of 3.20%, with a range of 3.04 to 3.36%. For crossbred, the subgroup SM for protein percentage recorded 3.34% (3.22 to 3.46%). Similar to the SM for milk fat percentage, the SM for protein percentage in crossbred cows was 4.4% higher than in foreign breeds. Overall, the SM for milk protein was 3.36%, with a range from 3.23 to 3.50%.

### Buffalo milk production and composition

**Fig. 6:**
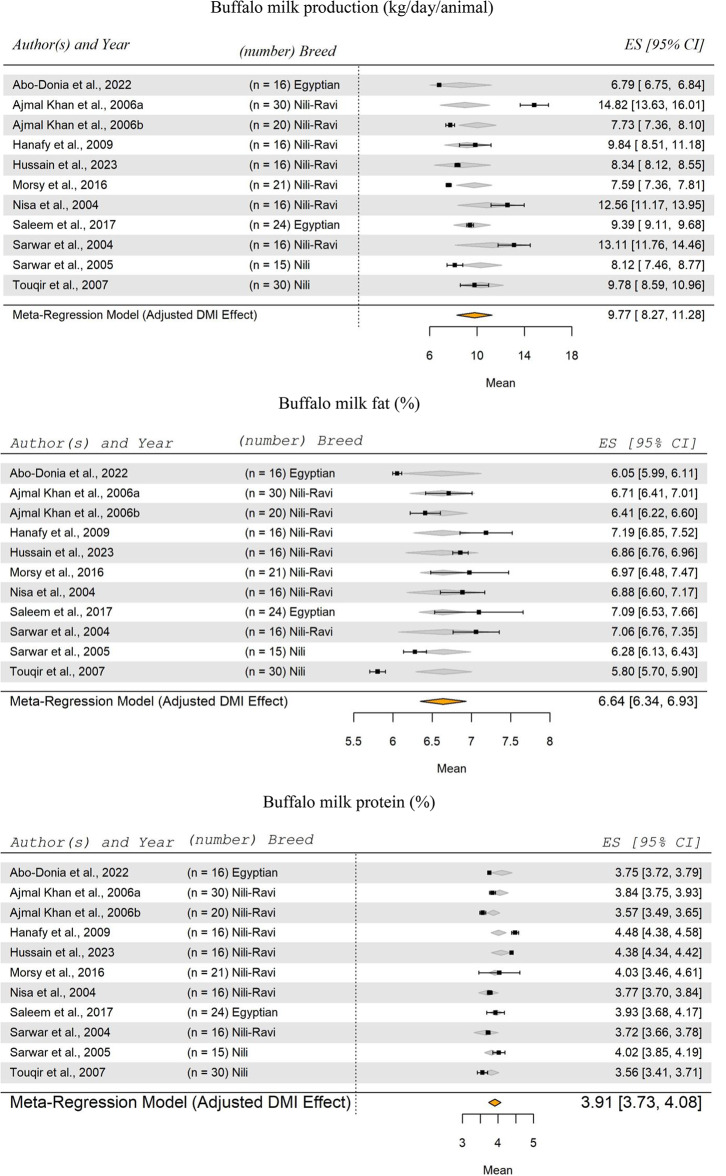
Forest Plot for Meta-Regression Analysis of buffalo milk yield (kg/day), milk fat (%), and protein (%). ‘n’ represents the number of animals used in each study, followed by the animal breed. The x-axis displays the z-statistic, which is the standardized mean (SM) for the studied parameter. The length of the horizontal lines represents the 95% confidence interval for the SM of studied parameter. The gray diamond represents the adjusted parameter to dry matter intake (DMI). The effect size (ES) value is the estimated parameter mean corrected for the number of animals used in each study, with a 95% confidence interval between practices

The meta-analysis of eleven studies included 220 animals (Abo-Donia et al. 2022; Ajmal Khan et al. 2006a, 2006b; Hanafy et al. 2009; Hussain et al. 2023; Morsy et al. 2016; Nisa et al. 2004; Saleem et al. 2018; Sarwar et al. 2004, 2005; Touqir et al. 2007), examined buffalo milk production, fat percentage, and protein percentage in warm-climate regions as shown in forest plots (Fig. 6). The effect of DMI on SM of milk production (kg/day/buffalo) was not significant (*P* = 0.289). The meta-analysis showed that the SM for buffalo milk production was 9.77 kg/day/head, with a range from 8.27 to 11.28 kg/day/head. Among river buffalo subspecies, the three breeds included in this study, Nili-Ravi, Egyptian, and Nili, are widely distributed and known for their efficiency in milk production (Borghese and Moioli 2016).

Regarding buffalo milk fat and protein, the meta-analysis results indicated that the effect of DMI on the SM of milk fat percentage and protein percentage was not significant (*P* = 0.931 and *P* = 0.100, respectively). The SM of milk fat for buffalo was 6.64%, with a range from 6.34 to 6.93%, while the SM for milk protein was 3.91%, with a range from 3.73 to 4.08%.

### Sheep and goat milk production and composition

**Fig. 7:**
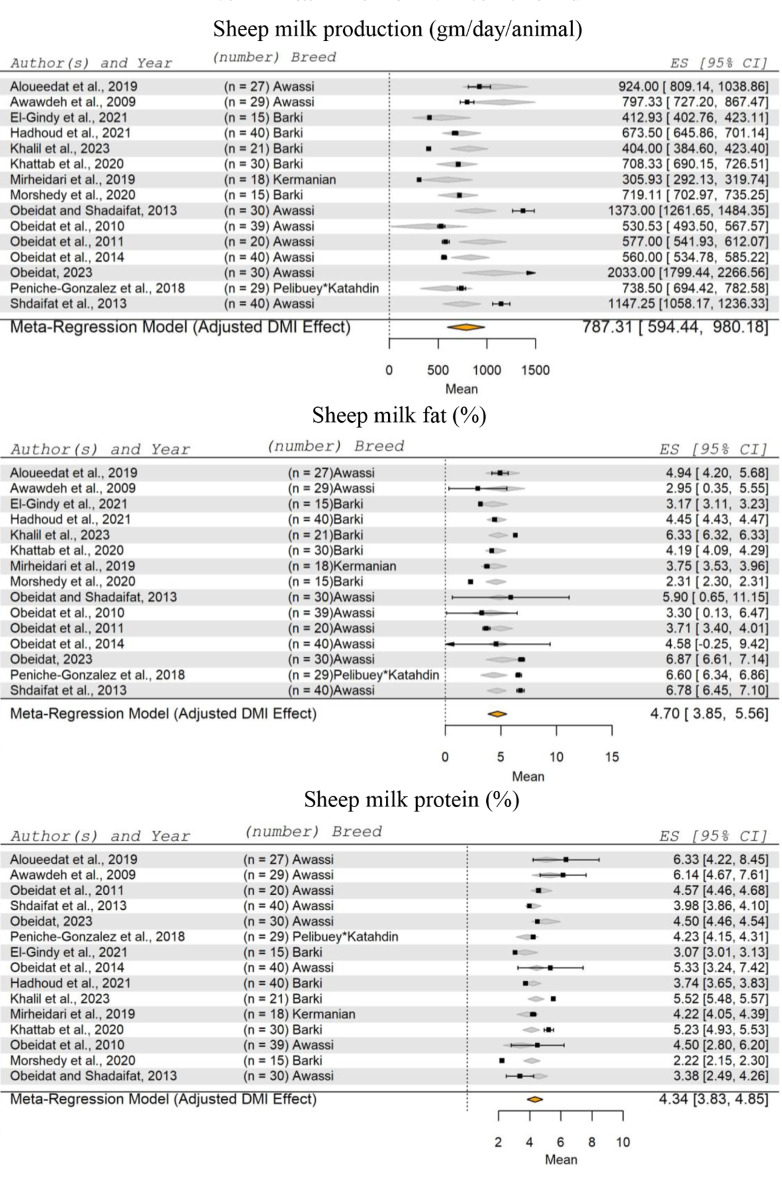
Forest Plot for Meta-Regression Analysis of sheep milk yield (kg/day), milk fat (%), and protein (%). ‘n’ represents the number of animals used in each study, followed by the animal breed. The x-axis displays the z-statistic, which is the standardized mean (SM) for the studied parameter. The length of the horizontal lines represents the 95% confidence interval for the SM of studied parameter. The gray diamond represents the adjusted parameter to dry matter intake (DMI). The effect size (ES) value is the estimated parameter mean corrected for the number of animals used in each study, with a 95% confidence interval between practices

The meta-analysis included fifteen studies (Aloueedat et al. 2019; Awawdeh et al. 2009; El-Gindy et al. 2021; Hadhoud et al. 2021; Khalil et al. 2023; Khattab et al. 2020; Mirheidari et al. 2019; Morshedy et al. 2020; Obeidat et al. 2010, 2011, 2014; Obeidat 2023; Obeidat and Shdaifat 2013; Peniche-Gonzalez et al. 2018; Shdaifat et al. 2013) on lactating sheep, comprising a total of 423 animal records (Fig. 7). The SM of milk production for sheep was 787.31 g/day/animal, ranging from 594.44 to 980.18 g/day/animal. The effect of DMI on SM of sheep milk production was significant (*P* = 0.0309).

Regarding the fat and protein percentages of sheep milk, the results showed that the SM for fat was 4.70%, and for protein, it was 4.34%. Milk fat percentage ranged between 3.85 and 5.56%, while milk protein percentage varied within range of 3.83–4.85%. Additionally, DMI had a non-significant effect on the SM of sheep milk fat and protein (*P* = 0.459 and 0.062, respectively).

**Fig. 8:**
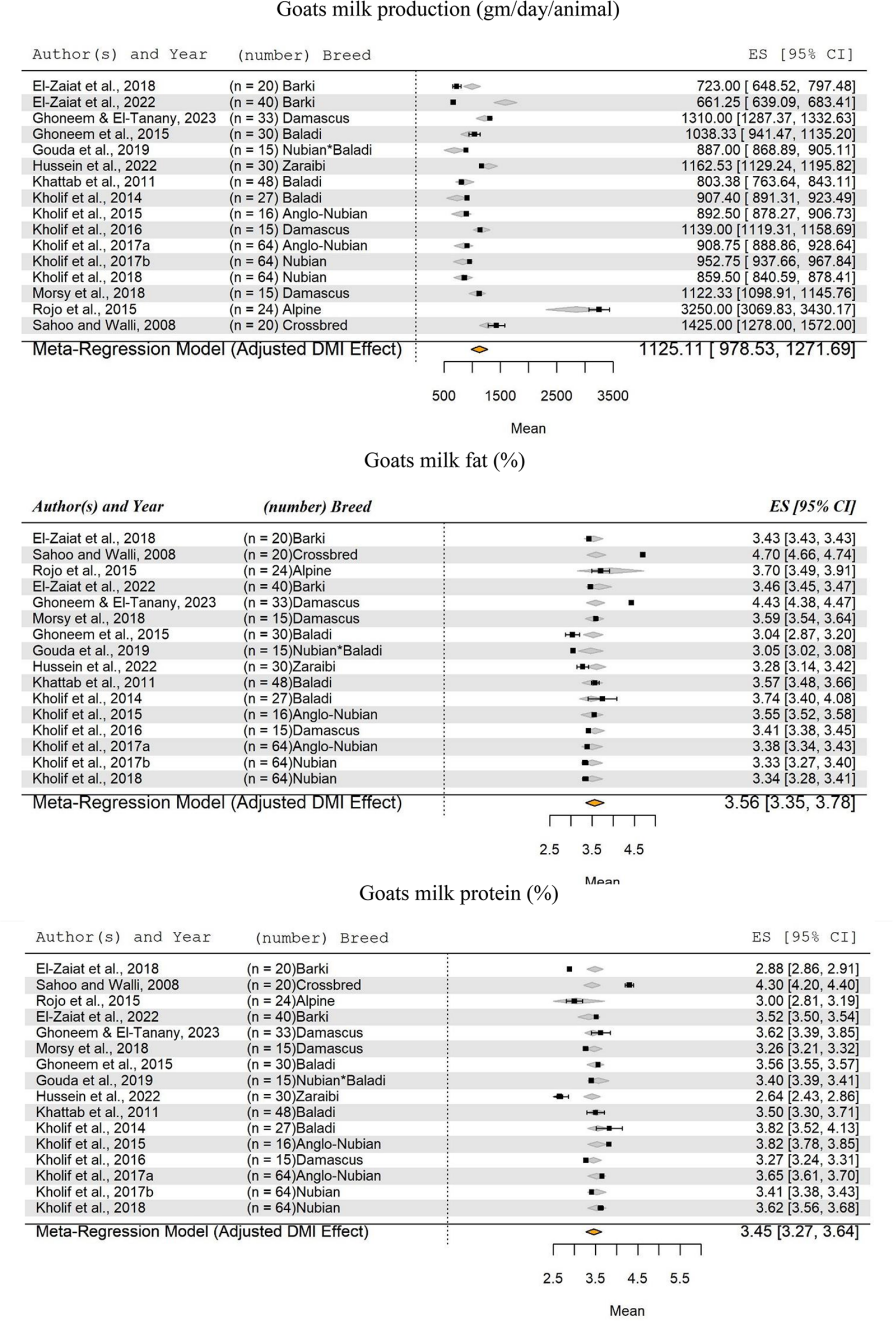
Forest Plot for Meta-Regression Analysis of goat milk yield (kg/day), milk fat (%), and protein (%). ‘n’ represents the number of animals used in each study, followed by the animal breed. The x-axis displays the z-statistic, which is the standardized mean (SM) for the studied parameter. The length of the horizontal lines represents the 95% confidence interval for the SM of studied parameter. The gray diamond represents the adjusted parameter to dry matter intake (DMI). The effect size (ES) value is the estimated parameter mean corrected for the number of animals used in each study, with a 95% confidence interval between practices

The current meta-analysis also included 16 studies (El-Zaiat et al. 2018, 2022; Ghoneem et al. 2015; Ghoneem and El-Tanany 2023; Gouda et al. 2019; Hussein et al. 2022; Khattab et al. 2011; Kholif et al. 2017a, 2018; Kholif et al. 2014, 2015, 2016, 2017b; Morsy et al. 2018; Rojo et al. 2015; Sahoo and Walli 2008) on goats milk production (g/day/head) and composition (fat and protein percentages) (Fig. 8). The DMI demonstrated a highly significant effect (*P* < 0.0001) on the SM of goats milk production. The results indicated that the SM for goat milk production was 1125.11 g/day/head, with a range from 978.53 to 1271.69 g/day/head. The analysis included 525 animal records from ten different breeds raised in warm-climate countries.

In relation to milk fat, the analysis indicated that the SM of goat’s milk fat percentage was 3.56 ± 0.21%, while the SM for protein percentage was 3.45 ± 0.18%. The effect of DMI on the SM of goats milk fat and protein percentages was found to be non-significant (*P* = 0.336 and 0.203, respectively).

## Discussion

A systematic review followed by a meta-analysis was conducted to quantitatively measure the milk production and composition of lactating cows, buffalo, sheep, and goats in warm-climate countries. Additionally, the study assessed the effect of dry matter intake (DMI) on milk production and composition across different species. According to our meta-analysis results, the DMI had a significant effect on cows’ milk production and protein percentage. Whereas its effect on the fat percentage of cow milk was insignificant. It has long been recognized that changes in milk production and composition, particularly in milk protein concentration, due to environmental effects (e.g., temperature-humidity index) are at least partly a result of the reduced DMI (Cowley et al. 2015). West (2003) and Hammami et al. (2013) reported that losses in milk yield can reach up to 0.96 kg/day, with a corresponding reduction in DMI of 0.85 kg, when dairy cows are exposed to heat stress.

The high milk production of crossbred cows is primarily due to the crossbreeding between hardy indigenous breeds and temperate breeds, such as Holstein or Holstein-Friesian, which are known for their high milk production. In contrast, indigenous cow breeds in tropical regions are characterized by traits such as heat tolerance, adaptability to seasonal changes, and the ability to cope with feed scarcity (Hernández-Castellano et al. 2019). Due to the limited number of research studies on native cow breeds; only three research papers were meet our criteria and were included in our meta-analysis. The primary reason for this is that indigenous breeds are commonly used as dual-purpose animals, providing both milk and meat, but with very low milk production (Djoko et al. 2003). However, the main challenge with expanding the number of foreign pure breeds is that they require more expensive investments in nutrition, housing, management, and heat stress mitigation systems (Michael et al. 2022). A summary of 23 studies showed a 2.4-fold increase in milk yield in tropical crossbreds with 50% Bos taurus bloodlines, compared to indigenous breeds (Galukande et al. 2013). Jenet et al. (2004) reported greater feed efficiency and a higher milk yield response to feed quality in the Holstein × Boran crossbred, compared to the indigenous Boran breed.

The higher milk fat and protein content observed in crossbred cows compared to foreign breeds in the present study in related to the effect of milk production. The decrease in milk fat and protein in temperate breeds like Friesian and Holstein may be associated with their high milk production (Harvatine et al. 2022). Our results confirm the impact of high temperatures in tropical and subtropical countries, which reduce milk protein concentration, casein number, and casein concentration (Cowley et al. 2015).

Regarding the buffalo results, the insignificant effect of DMI on the SM of milk production could be due to the low DMI variance between lactating buffalo breeds included in this study. Purcell et al. (2016) reported that the method of concentrate feeding had no effect on the performance of high-producing females in early to mid-lactation when all females were offered the same amount of concentrate in addition to a basal diet provided ad libitum. This contrasts with the findings of Gaafar et al. (2009) who demonstrated higher milk yield in lactating buffaloes fed ration consisting of 40% concentrate and 60% roughages on DM basis. Lawrence et al. (2017) reported that increasing the total amount of concentrate offered led to higher total DM and energy intake, which resulted in increased milk production. Similarly, Habib et al. (2020) found that the additional of concentrate to the existing feed of lactating buffaloes can increase milk yield, although no significantly difference in milk compositions among the buffaloes were observed. Samad et al. (2022) also reported that milk production increased with higher DMI in inter-species lactating dairy buffaloes.

In a study by Sarwar et al. (2009), it was reported that composition of buffalo milk is affected by nutrient intake and quality. According to Wahid and Rosnina (2011), feeding buffaloes concentrate can increase the milk fat content by up to 15%, as buffalo release extra fat into the milk. Our results are align with those of Abdel-Hamid et al. (2023), who compared the milk composition of three buffalo breeds and found that the average fat percentage for Nili-Ravi buffalo was 6.84%. Additionally, a recent meta-analysis by Medrado et al. (2021) estimated genetic parameters for economic traits in buffalo and found negative correlations between milk yield and fat percentage (-0.21) and milk yield and protein percentage (-0.20).

Regarding the small ruminant results, there was a significant effect of DMI on the SM of milk production in ewes and goats. This finding may be accurate, particularly during the early stage of lactation, when the mother nutritional requirements for milk production are high (Hassoun et al. 2021). Meanwhile, the DMI is weakly correlated with milk yield in mid- and late phases of lactation period. The SM value for ewes’ milk production in the present meta-analysis was lower than those reported in a recent meta-analysis by Chay-Canul et al. (2020) for two tropical hair sheep breeds, Pelibuey and Katahdin. The milk production values for Pelibuey and Katahdin were 1.44 and 1.77 kg/d/animal, respectively, compared to 0.98 kg/d/animal, which was the highest value for SM range in current meta-analysis. Additionally, a slight decrease in milk fat and protein content was observed compared to previous study. This variance may be attributed to intensive selection for milk production in Pelibuey and Katahdin breeds. Furthermore, this discrepancy may be related to animals’ response to temperature effects, as temperatures exceeding the upper critical threshold can negatively impact the productive variables of lactating ewes (Sevi and Caroprese 2012). The chemical composition of small ruminants’ milk is more responsive to the dietary composition than that of cattle (Olvera-Aguirre et al. 2020). Meanwhile, the range of sheep SM of milk fat% (3.85 to 5.56%) was consistent with several studies that were not included in the present meta-analysis (Babiker et al. 2017; Chay-Canul et al. 2020; Olvera-Aguirre et al. 2020). Additionally, in the study by Ferro et al. (2017), the milk fat percentages of lactating ewes for the three breeds, Awassi, East Friesian, and fat-tailed, were recorded as 5.87, 5.95, and 5.26%, respectively. These results were consistent with the findings of the current meta-analysis. However, the percentages of milk protein for these breeds were higher than the current meta-analysis results.

The SM of goat milk production aligns with the research conducted by Knights and Garcia (1997), who reviewed the lactation performance of goat breeds from tropical and subtropical regions in tropical and subtropical countries. The daily yield values in their study were consistent with the current meta-analysis range of 1.13 ± 0.15 kg/day/head for SM of milk production for Beetal, Damascus, Kilis, and Anglo-Nubian goat breeds, but contrasted with those for Jamunapari, Boer, Nubian, Saanen, and British Alpine. The range of SM for goat milk fat percentage followed a similar trend to that previously reported in other studies on goats raised in hot climate (AL-Suwaiegh 2016; Olvera-Aguirre et al. 2020). The SM range for milk protein percentage matches the results from Devendra (1980) for several goat breeds, including Beetal and Anglo-Nubian. Furthermore, the protein percentage range aligns with the findings of Ferro et al. (2017) in their review of the Anglo-Nubian, La Mancha, Malaguena, and Maltese breeds, which reported milk protein percentages of 3.3%, 3.34%, 3.40%, and 3.48%, respectively.

In a recent global meta-analysis by Akshit et al. (2024) that assessed goat milk contents across 82 references regardless the geographical origin, spanning from 1975 to 2023, the SM for protein content in goat milk was 3.52 ± 0.16%, compared to 3.45 ± 0.18% in the present meta-analysis. Conversely, the SM for milk fat was 4.13 ± 0.43%, which is higher than current result of 3.56 ± 0.21%. This variance could be related to the high heterogeneity of milk fat content among the different breeds included in the analysis by Akshit et al. (2024). In hot weather, goats reduce their feed intake to minimize body heat production. This reduction in feed intake results in decreased glucose uptake by the mammary gland, which consequently affects milk composition (Scano and Caboni 2022). Goat breeds in hot climate countries are well adapted to high temperature-humidity index (THI) levels. El-Tarabany et al. (2017) reported no significant differences in the milk fat or protein content of Egyptian Baladi goats at varying THI levels up to 80. In contrast to this finding, a study conducted in temperate regions by Kljajevic et al. (2018) found a negative correlation between THI and the chemical characteristics of Saanen goat milk in Serbia.

According to our best knowledge, this meta-analysis study is the first to focus on estimating milk production and quality in cattle, buffalo, sheep, and goats in warm-climate regions. The random effects model used in the current meta-analysis was essential due to the large heterogeneity among studies of milk production in cows. The current findings on milk production and composition in the tropics and subtropics are crucial for improving the performance of the studied livestock species. Moreover, comparing milk productions per animal across different ruminant species with that in other countries could help identify the potential for crossbreeding to enhance milk production in hot climate countries.

## Data Availability

The datasets generated and/or analyzed during the current study are available from the corresponding author on reasonable request.
