# Correction to: Milk production and composition in warm-climate regions: a systematic review and meta-analysis

**DOI:** 10.1007/s11250-024-04247-w

**Published:** 2024-12-03

**Authors:** Mohamed Rashid, Hadeer M. Aboshady, Rania Agamy, Harry Archimede

**Affiliations:** 1 Regional Center for Food and Feed, Agricultural Research Center, Giza, 12619 Egypt; 2 Animal Production Department, Faculty of Agriculture, Cairo University, Giza, 12613 Egypt; 3 Agroecology, Genetic and Tropical Livestock Farming System, INRAE, Petit-Bourg, Guadeloupe 97170 France

**Correction to: Tropical Animal Health and Production**

10.1007/s11250-024-04214-5

The original version of this article inadvertently contained several mistakes that are herein corrected. Authors would like to present their apologies for the oversight.

The corrections are as follows:

In Figs. 6 and 8, there are missing parts as mentioned below:

1. Figure 6: first part need to mention "Buffalo milk production (kg/day/animal)".
2. Figure 8: first part of the figure is missing "Goats milk production (gm/day/animal)" and the title of the second part of the same figure "Goats milk fat (%)".

The corrected Figs. 6 and 8 are given as follows:

Figure 6:

**Fig. 6:**
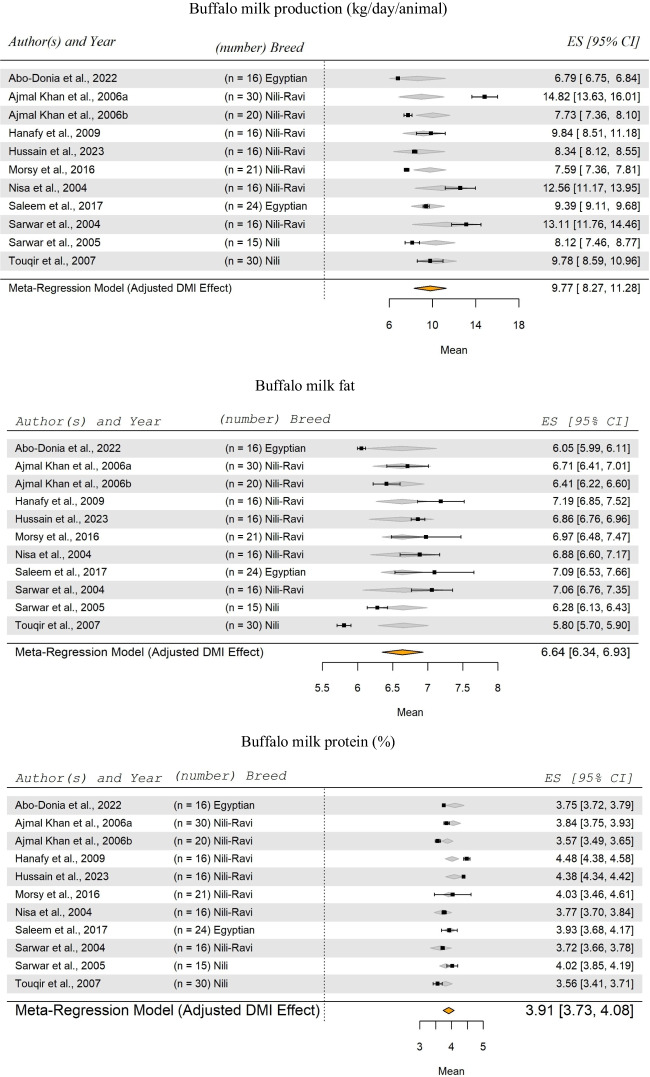
Forest Plot for Meta-Regression Analysis of buffalo milk yield (kg/day), milk fat (%), and protein (%). 'n' represents the number of animals used in each study, followed by the animal breed. The x-axis displays the z-statistic, which is the standardized mean (SM) for the studied parameter. The length of the horizontal lines represents the 95% confidence interval for the SM of studied parameter. The gray diamond represents the adjusted parameter to dry matter intake (DMI). The effect size (ES) value is the estimated parameter mean corrected for the number of animals used in each study, with a 95% confidence interval between practices

Figure 8:

**Fig. 8:**
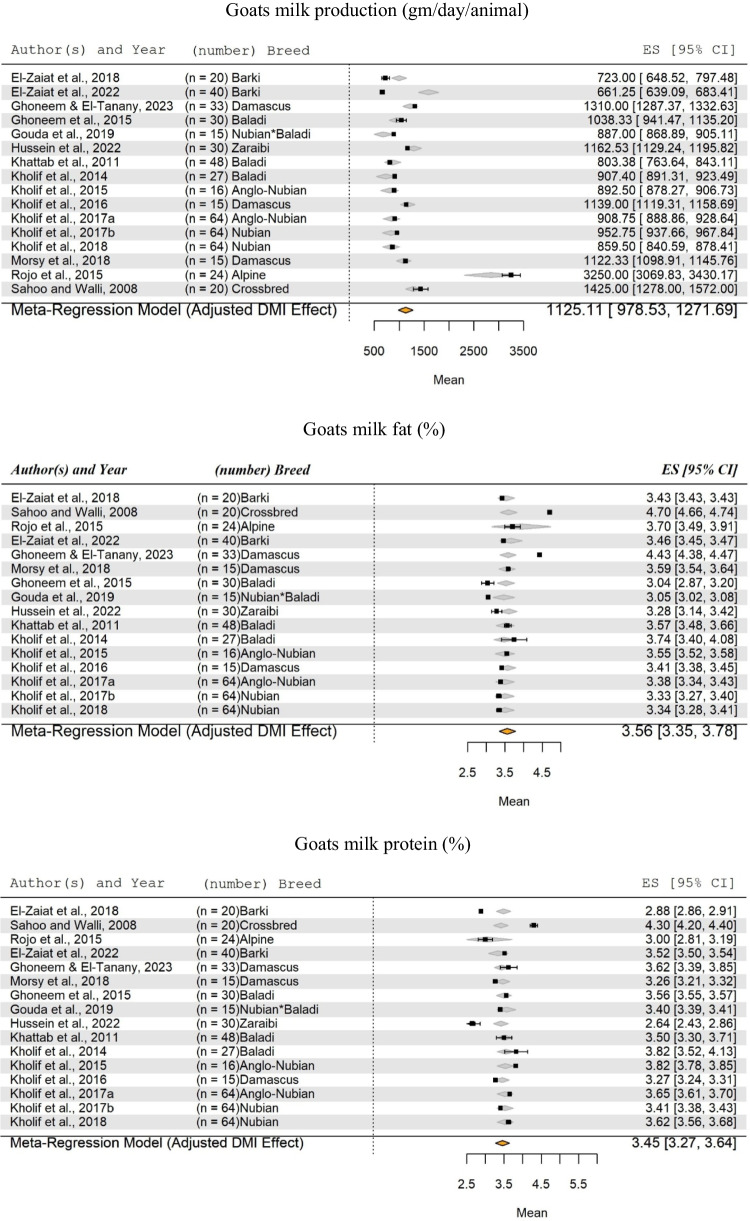
Forest Plot for Meta-Regression Analysis of goat milk yield (kg/day), milk fat (%), and protein (%). 'n' represents the number of animals used in each study, followed by the animal breed. The x-axis displays the z-statistic, which is the standardized mean (SM) for the studied parameter. The length of the horizontal lines represents the 95% confidence interval for the SM of studied parameter. The gray diamond represents the adjusted parameter to dry matter intake (DMI). The effect size (ES) value is the estimated parameter mean corrected for the number of animals used in each study, with a 95% confidence interval between practices

The original article has been corrected.

